# Investigating the validity of low-cost technologies for the assessment of jumping-based performances in people with patellofemoral pain

**DOI:** 10.17159/2078-516X/2025/v37i1a20301

**Published:** 2025-05-15

**Authors:** G Deysel, M van Aswegen, M Kramer

**Affiliations:** Physical Activity, Sport, and Recreation (PhASRec) Research Focus Area, North-West University, Potchefstroom, North West Province, South Africa

**Keywords:** jump, unilateral, video analysis

## Abstract

**Background:**

Patellofemoral pain (PFP) is prevalent across various age and activity groups and poses a risk for developing patellofemoral osteoarthritis. Since load on the patellofemoral joint is greatest during jumping manoeuvres, validating affordable measuring instruments to extract jumping-related variables is important for guiding rehabilitation.

**Objectives:**

To evaluate the validity of low-cost devices against ‘gold standard’ force plates during jumping and to quantify differences in kinematic variables between low-cost devices and across different groups (PFP vs. Control).

**Methods:**

A repeated-measures design of between- and within-subject factors was used. Thirty-two participants (Control: n=16; PFP: n=16) volunteered for the study. Single leg drop jump variables were validated using force plates and 3D motion capture (mocap) as the criterion standards against the MyJump2 and Tracker software applications as the reference standards.

**Results:**

Good-to-excellent correlations were evident across all variables when comparing the force plates to MyJump2 (r=0.83–0.97) and Tracker (r=0.83–0.89) applications. Tracker was not significantly different from force plates or mocap for jump height (p=0.130) and flight time (p=0.230), but overestimated contact time for both groups (control group [p<0.001] and PFP group [p=0.007]). MyJump2 was not significantly different from force plates regarding contact time in the PFP group (p=0.500) but showed significant differences for the other parameters (p<0.001).

**Conclusion:**

Both Tracker and MyJump2 applications show promise as alternatives to laboratory-grade equipment, with MyJump2 emerging as the top low-cost tool.

Patellofemoral pain (PFP) is a prevalent condition that affects approximately 23% of adults^[1]^ and up to 40% of recreational runners.^[2]^ Given that people with PFP experience more physical, emotional, and social limitations during sports activities and activities of daily living^[3]^ and that PFP may persist over several years, leading to patellofemoral osteoarthritis later in life^[4]^, early identification and rehabilitation is important.

More specifically, PFP is worsened by actions that load the patellofemoral joint during weight-bearing flexion-based activities such as running, stair climbing, jumping, or squatting. More explicitly, loads on the patellofemoral joint are highest during jumping manoeuvres (i.e. 9–11 multiples of body weight).^[5]^ Therefore, it can be helpful to evaluate jumping tasks to better determine the possible causes of pain and improve treatment selection and monitoring in people with PFP.

Despite a large body of research dedicated to PFP, the recurrence rates remain high (up to 40%).^[6]^ Furthermore, it should be noted that the “gold standard” instruments for measuring dynamic movements like jumping include three-dimensional (3D) motion capture (mocap) systems^[7]^ and force plates.^[8]^ Such systems are typically not affordable by practitioners because costs exceed $15 000 (force plates) or $100 000 (mocap). Therefore, a need exists to assess and validate lower-cost alternatives for practitioners to determine if such substitutes meet the criteria for clinical use. The *MyJump2* application (app) is a validated low-cost cell phone tool used within the sporting population but has limited research on the validation within a rehabilitative environment.^[9]^ The *Tracker Motion Analysis* software is also a validated computer application for physics experiments^[10]^ but has not been used, or validated for use within rehabilitation settings.

Therefore, the study’s objectives were: i) to evaluate the validity of low-cost tools during a unilateral jumping task relative to ‘gold standard’ equipment, and ii) to assess jumping-based between-group differences in kinematic variables using low-cost devices.

## Methods

### Study design

This study used a repeated measures design comprising a combination of between- and within-subject factors.^[11]^ The between-subjects independent variable was group allocation (control group and PFP group), where group assignment was not randomised due to the presence or absence of injury. Instead, the control group was used to compare data from the PFP group to evaluate testing consistency. The within-subjects independent variables included flight time and jump height (repeated measures), and extremity (injured [or non-dominant] and uninjured [or dominant] lower limb).

### Participants

A minimum sample size of 24 was calculated based on a repeated measures analysis of variance (rmANOVA) design that incorporated (i) a within-between interaction, (ii) a moderate effect size (f=0.25), (iii) a type-I error rate of 5% (α=0.05), (iv) a type-II error rate of 20% (β=0.20), (v) two groups (control [n=17] and PFP [n=17]), (vi) two repeated measurements, and (vii) a minimum expected correlation of 0.50 among repeated measurements.^[12]^ A total of 35 participants volunteered for the study, of which 17 were part of the control group (female [n=10] and male [n=7]) and 18 were part of the PFP group (female [n=15] and male [n=3]. Accounting for a potential drop-out of 20%, the minimum sample size for adequate statistical power was 29 participants. Three participants were lost to follow-up, two in the PFP group (female [n=1] and male [n=1]), and one in the control group (male [n=1]) due to incomplete data, resulting in a final sample size of 32 participants.

The inclusion criteria for the PFP group consisted of the following: (i) aged 18 to 35 years, (ii) could be male or female, (iii) had to have retropatellar and/or peripatellar pain aggravated by at least one activity that loads the patellofemoral joint during weight bearing on a flexed knee (squatting, stair climbing, jogging/running, and hopping/jumping), and (iv) had to participate in some form of rehabilitation program. The control group had the same inclusion criteria except for points (iii) and (iv).

The exclusion criteria for both groups consisted of the following: participants should not (i) have had previous patellar dislocation or subluxation, (ii) have had previous injury or surgery to the knee, and (iii) have had recent (within the last six months) injury to the lower limbs (e.g. Achilles tendinopathies, ankle sprain, etc.).

### Ethical clearance

This study gained ethical approval from the North-West University Health Research Ethics Committee (NWU-HREC), North-West University, Potchefstroom, South Africa, on 20 Nov 2022, with ethics number NWU-00163-22-A1. All participants completed the informed consent forms prior to participation. All ethical procedures conformed to the requirements of ethical conduct set forth in the Declaration of Helsinki.

### Instruments and procedures

A general demographic questionnaire was completed first to obtain the participant’s contact details, e-mail address, age, sex, involved limb, dominant limb, previous injuries and PFP symptoms. Body mass and stature were then measured to standardise the effect of jump-landing forces across individuals. Body mass was measured in minimal clothing and barefoot with an electronic scale (Seca 874, Seca, Germany) to the nearest 0.1 kg and stature with a portable stadiometer (Holtain Ltd., U.K.) to the nearest 0.01 m. Participants then completed a 10-minute warm-up on a cycle ergometer (Wattbike Pro, Wattbike Ltd, Nottingham, UK) at a low resistance and a comfortable speed (rating of perceived exertion [RPE] < 2 on the modified Borg scale) before the testing for optimal muscle performance and reduced risk of injury.

The evaluation of the drop jump was then completed on a tri-axial force plate (AMTI, Watertown, MA, USA), and 3D motion capture (mocap) to track the movements of markers (Qualisys Inc., Gothenberg, Sweden). Force plates were used to measure ground reaction force at a sampling frequency of 2000 Hz, whereas the mocap system was used to record marker trajectories at a sampling frequency of 200 Hz. The low-cost alternatives consisted of the following: (i) the MyJump2 cell phone application (My Jump Lab, v1.1.2, Balsalobre C.) and (ii) the Tracker computer software (Tracker, v6.1.5, Brown D. et al.). A Samsung Galaxy Note 10 cell phone with a 1080×2280 pixels resolution and a 12 + 12 + 16-megapixel camera recorded video data at a sampling frequency of 60Hz, and was mounted on a tripod 2.0-m from the lateral edge of the force plates to capture video of the jumps during testing. The jumps were recorded from the side of the jumping leg to ensure that marker trajectories were always visible. A Bluetooth remote was used to start and stop the video. Reflective markers were placed on the lateral malleoli of the ankles and the greater trochanter of the femur for tracking within the mocap system and Tracker software. Participants completed five familiarisation trials on each leg before the test. Thereafter participants were granted two minutes of rest to ensure complete recovery before starting with the test.

The single-leg drop jump was used since it evaluates eccentric loading of the lower extremities to a greater extent, thereby more likely stressing the patellofemoral joint in order to accentuate inherent differences between groups.^[13]^ The single-leg drop jump was performed by standing with both feet on a 0.25m platform with the hands on the hips. On the queue “drop” the participants dropped down. They landed on the jumping leg on the centre of the force plate and performed a maximal effort vertical jump immediately upon landing. The participants were instructed to only “drop” off the step (i.e. no jumping off), which was verified by visual inspection of the marker trajectories on the mocap system. Participants were permitted one minute rest between repetitions, and three repetitions of the single-leg drop jump on each leg were retained for analysis, of which the best (according to jump height) was used in the final assessment. Trials were discarded and subsequently repeated if: (i) a noticeable ‘jump’ was performed off the step, (ii) participants lost balance on landing, and/or (iii) jumped/stepped off the force platform following the second landing. The raw force-time data were exported to Matlab (The Mathworks, MA, USA) for processing. Force data were smoothed using a 4th-order, zero-lag Butterworth filter with a cutoff frequency of 30Hz. For each single-leg drop jump trial, the instant of landing and take-off was classified when the force values exceeded 30N, which could then be used to characterise flight time (FT) or contact time (CT).

The following parameters were recorded for the jumping trials from the force plate:

1.
$\text{Jumping height }(\text{m}):\frac{1}{8}\cdot g\;({kgms}^{-2})\cdot {FT}^{2}(s)$.
2. Flight time (FT [s]): time between take-off (when the force reading dropped below 30 N) to foot contact on the second landing (when the force reading exceeded 30 N).
3. Contact time (CT [s]): time between first landing from step (when the force reading exceeded 30 N) to take-off (when the force reading dropped below 30 N).

The following parameters were recorded for the jumping trials from the video files:

1. Jumping height (m): difference between peak marker height and marker position at peak plantarflexion before take-off.
2. Flight time (s): time period between marker height at peak plantarflexion before take-off and when the marker was at the same height before the second landing.
3. Contact time (s): time period between marker height at peak plantarflexion at the instant of landing and when the marker was at the same height before take-off.

The MyJump2 Application and Tracker software used the video data to extract the parameters of interest (e.g., jump height, flight time, contact time).

**Fig. 1:**
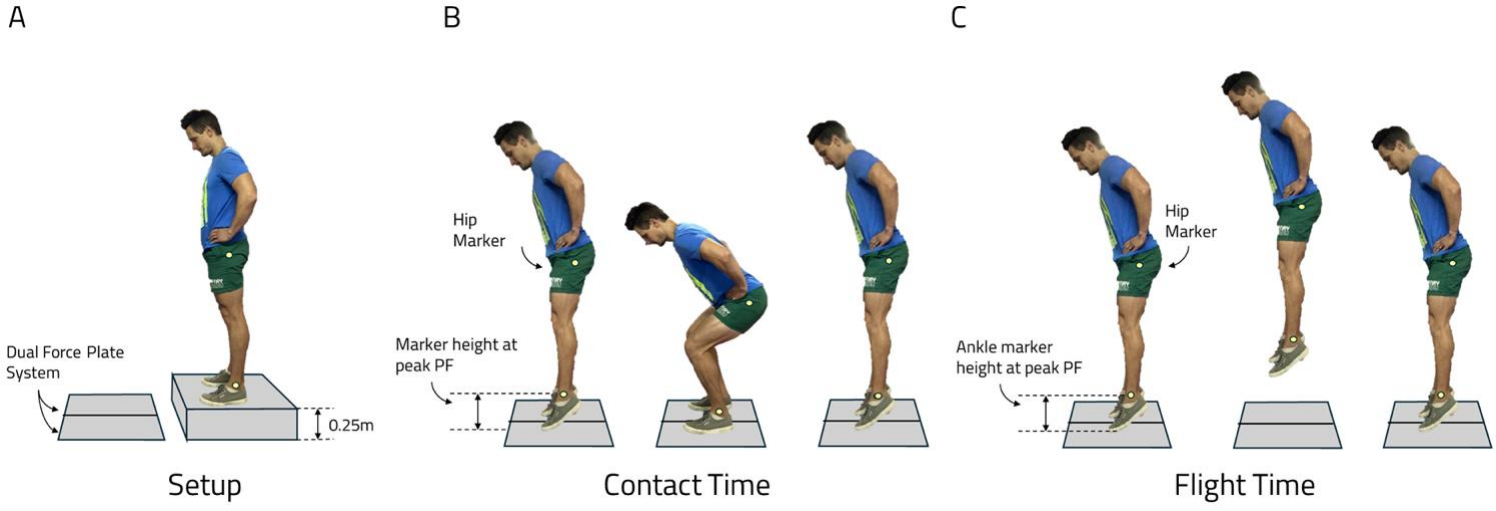
Example of experimental setup where reflective markers were placed at the greater trochanter and lateral malleolus for tracking (yellow dots). Panel A: indicates the participant’s position in preparation for the drop jump. Panel B: indicates the position of the participants in which contact time was evaluated. Panel C: indicates the participant’s position in which flight time was evaluated.

A visual representation of the experimental setup and data extraction for flight time and contact time are shown in Figure 1.

### Statistical analyses

All statistical analyses were completed using R (RStudio, version 2023.06.1, build 524, Posit Software, PBS [Windows]). Data were evaluated for normality using the Shapiro-Wilk test from the *stats* package, whereby deviations from normality were accepted when p<0.05. The *stats Expressions* package (version 1.6.1) was used to appraise between-group differences for the kinetic and kinematic data using an independent t-test. The *ggstatsplot* package (version 0.11.0) was used to complete the rmANOVA (Fisher) to evaluate the mean differences in parameter estimates across the different devices. The post-hoc Holm correction was used to adjust for multiple pairwise comparisons. The partial omega squared (ω_p_^2^) was used as a measure of the standardised effect size for the rmANOVA and was qualitatively interpreted as: very small: <0.01; small: 0.01–0.059; medium: 0.06–0.14, and large: >0.14. The *stats* package was used to evaluate the validity of parameters across the different devices using a linear regression model where the Pearson correlation coefficient (r), coefficient of determination (r^2^), standard error of the estimate (SEE), and the slope of the regression lines were assessed. The magnitude of the correlation coefficients were qualitatively interpreted as follows: negligible: 0.00–0.09; weak: 0.10–0.39; moderate: 0.40–0.69; strong: 0.70–0.89; and very strong: 0.90–1.00.^[14]^ The coefficient of determination values were used as a measure of whether the low-cost device can be compared with the reference device and were interpreted as follows: 0 = no comparison; <0.19 = very weak comparison; 0.19–0.32 = weak comparison; 0.33–0.67 = moderate comparison; >0.67 = substantial comparison; and 1 = perfect comparison.^[15]^ The systematic bias between devices (e.g. Tracker and MyJump2 ) and the criterion measure (e.g. force plate, mocap) was evaluated using Bland-Altman plots.^[16]^ For both the regression and Bland-Altman analyses, the point estimates were appraised for outliers using Cook’s distance (calculated using the *olsrr* package [version 0.6.1]), where potential outliers (PO) were flagged when the Cook’s distance exceeded a given threshold calculated as: 4/n (where n is the number of observations).^[17]^

## Results

### Validity

**Fig. 2:**
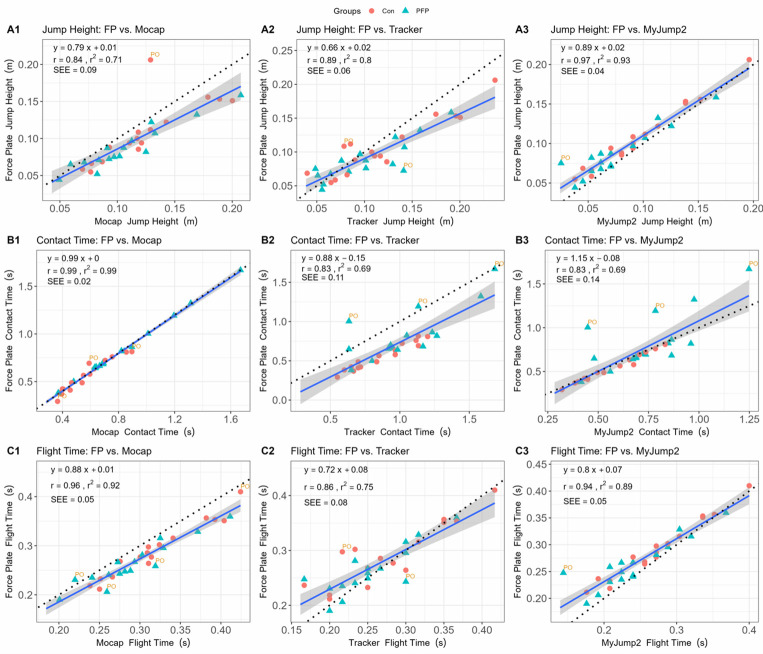
Regression results for the association between parameters derived from the force plate (gold standard) and reference methods. Panel A shows the association of the jumping height between the force plate and Qualisys (mocap) marker (A1), Tracker (A2), and My Jump 2 App (A3). Panel B shows the association of contact times between the force plate and Qualisys (mocap) marker (B1); Tracker (B2), and My Jump 2 App (B3). Panel C shows the association for flight times between the force plate and Qualisys (mocap) marker (C1), Tracker (C2), and My Jump 2 App (C3). Con, control group (indicated with dots); mocap, motion capture; PFP, patellofemoral pain group (indicated with triangles); PO, potential outlier; SEE, standard estimate of error.

The results for the regression analyses, which provide information related to the validity of the derived kinematic parameters across the different devices, are shown in Figure 2. Generally, there were strong-to-very strong associations (r=0.83–0.99) between all parameters across all systems. The strongest associations were between the force plate and mocap systems, followed by the MyJump2 App, and finally the Tracker software.

**Fig. 3:**
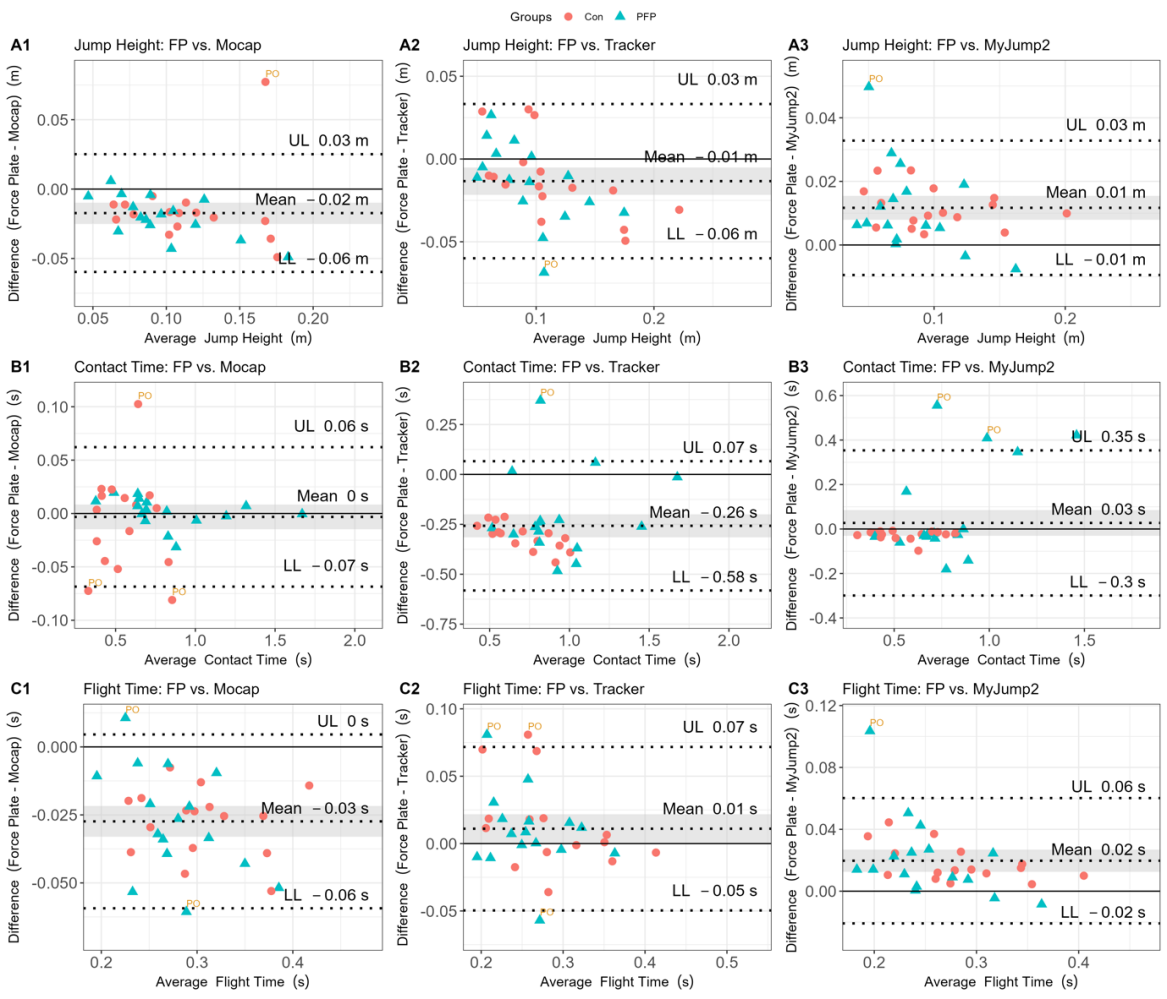
Bland-Altman results for evaluating differences between parameter estimates derived from the force plate (gold standard) and reference methods. Panel A shows the bias and limits of agreement (LoA) for jumping height between the force plate and Qualisys (mocap) marker (A1); Tracker (A2), and My Jump 2 App (A3). Panel B shows bias and LoA for contact times between the force plate and Qualisys (mocap) marker (B1); Tracker (B2), and My Jump 2 App (B3). Panel C shows bias and LoA for flight times between the force plate and Qualisys (mocap) marker (C1); Tracker (C2), and My Jump 2 App (C3). FP, force plate; Con, control group (indicated with dots); mocap, motion capture; PFP, patellofemoral pain group (indicated with triangles); PO, potential outlier; SEE, standard estimate of error.

The results for the systematic bias between each of the low-cost devices for each of the derived kinematic parameters are shown in Figure 3.

Compared to force plates, marker tracking on the mocap system generally over-predicted jump height (M_diff_=0.1 to 0.02m), whereas the MyJump2 App under-predicted jump height. Estimations of contact time were almost identical between the mocap system and the MyJump2 App, whereas the Tracker software over-predicted contact duration. Flight time was marginally higher for the marker tracking using mocap, but lower for the Tracker and MyJump2 systems.

### Kinematic data

**Fig. 4:**
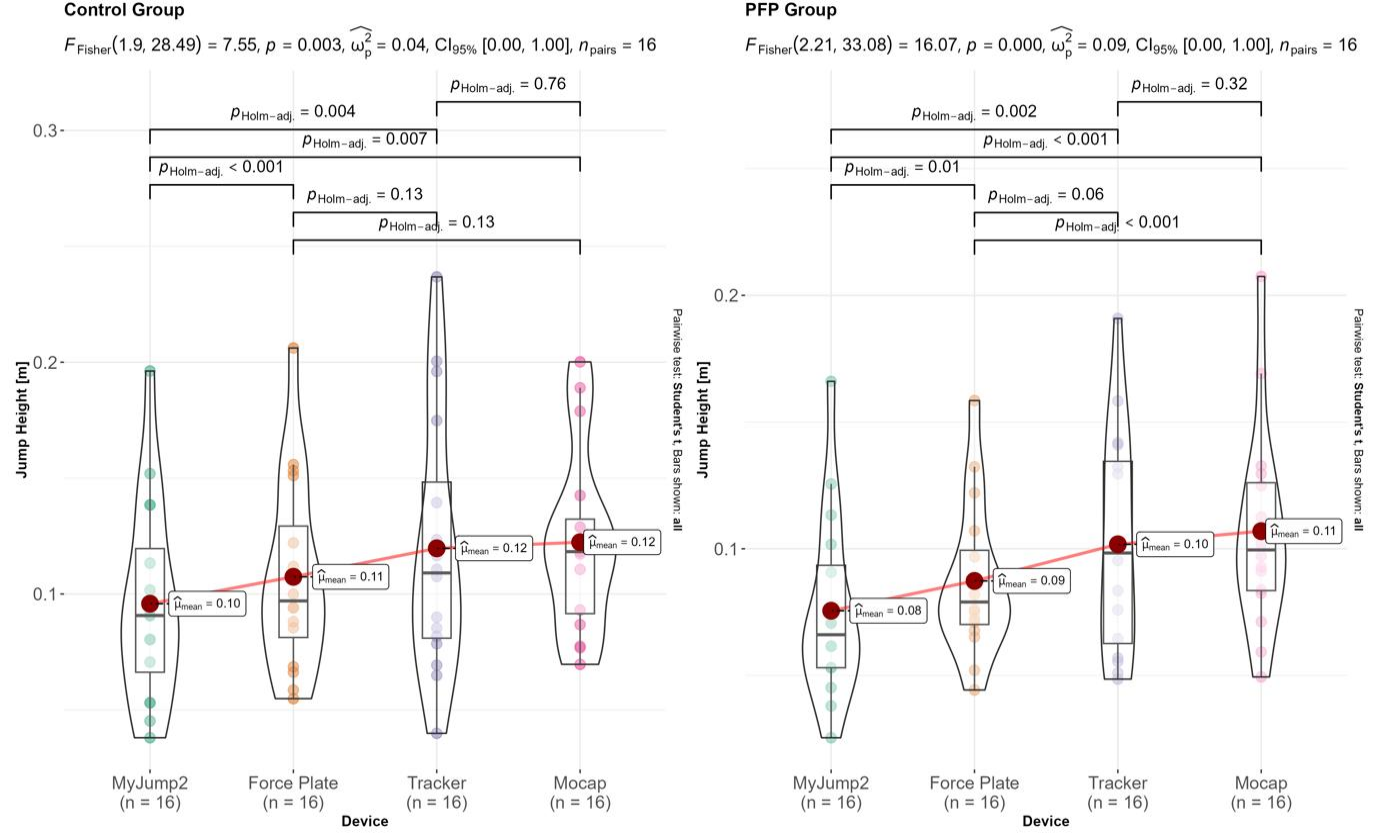
Inferential results for within-group differences of jump height. Jumping height is shown for the control (left) and PFP (right) groups respectively. Con, control group; PFP, patellofemoral pain.

**Fig. 5:**
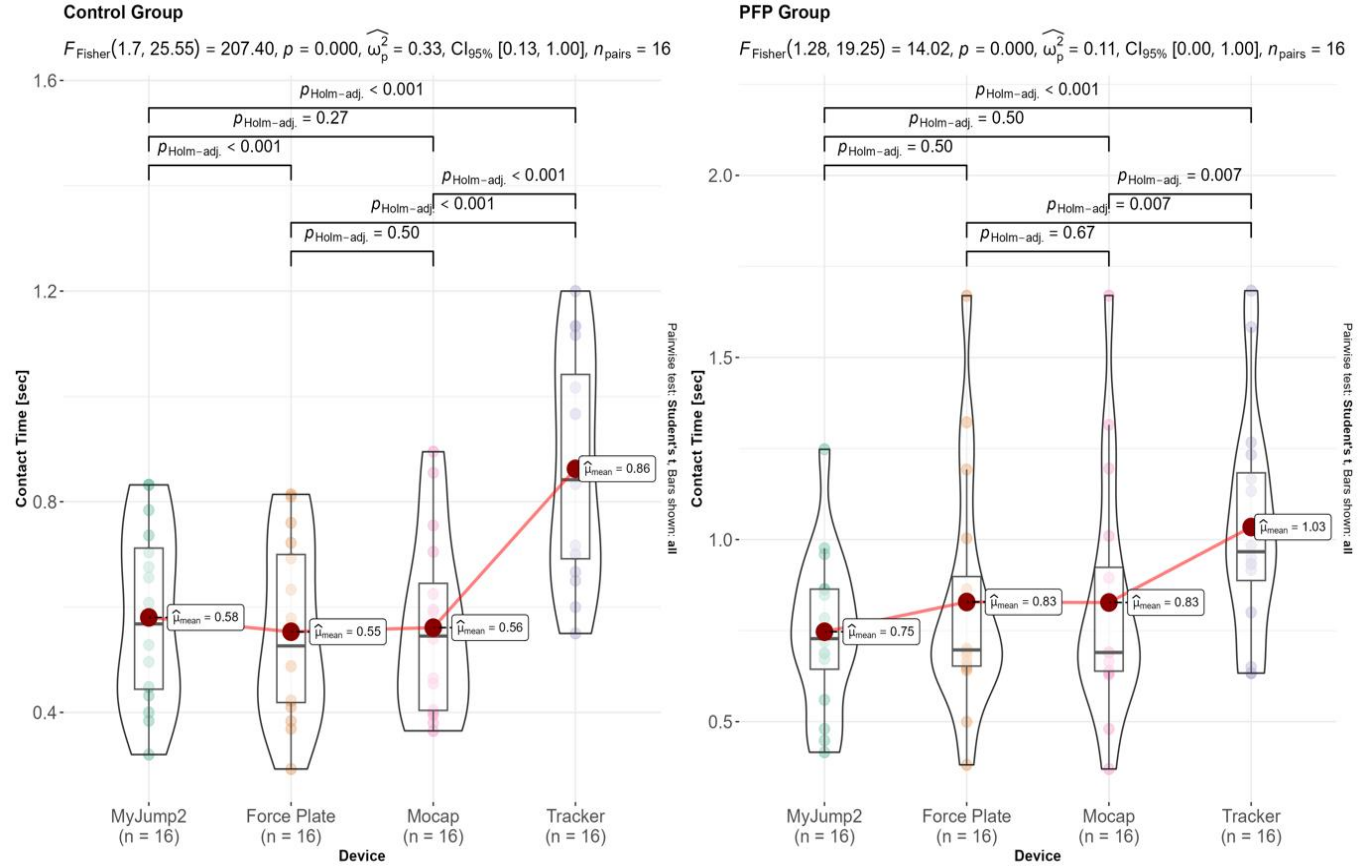
Inferential results for within-group differences of contact time. Contact time is shown for the control (left) and PFP (right) groups respectively. Con, control group; PFP, patellofemoral pain group.

**Fig. 6:**
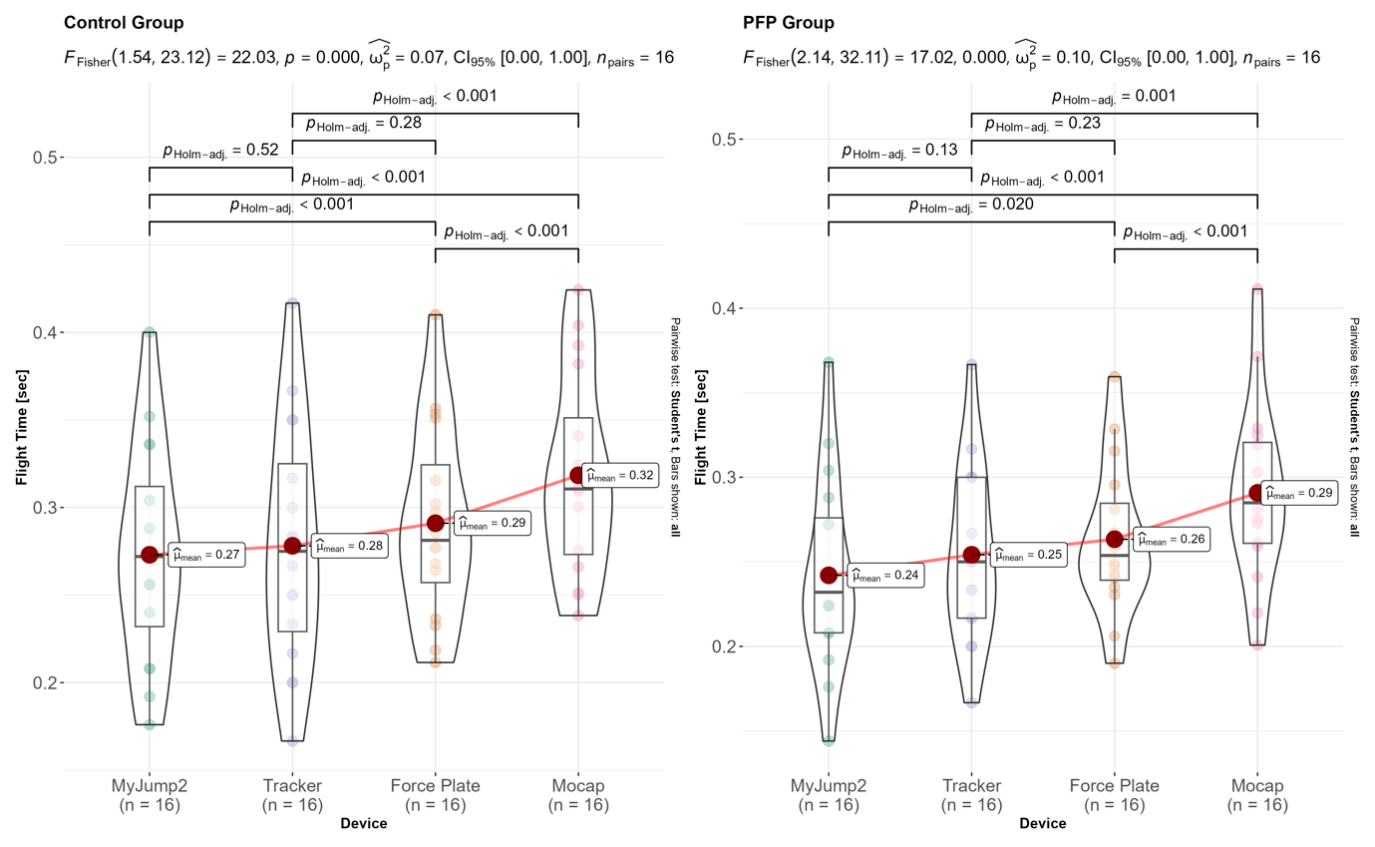
Inferential results for within-group differences of flight time. Flight time is shown for the control (left) and PFP (right) groups respectively. Con, control group; PFP, patellofemoral pain group.

The results for within-group differences across the different devices for jumping height, contact time, and flight time are shown in Figures 4–6. The overall inferential results for the rmANOVA are shown at the top of each panel, whereas the data for the post-hoc analyses are shown within each panel. Only statistically significant differences between equipment are indicated with horizonal block brackets and the adjusted p-values using the Holm correction.

## Discussion

The present study revealed that low-cost technologies could be used to extract kinematic parameters such as jump height, contact time, and flight time but exhibited marginal discrepancies when compared to criterion methods, which may preclude the interchangeability of parameters across devices.

Our results generally indicated good-to-excellent agreements between the force plate and alternative devices (r=0.83–0.99 and r^2^=0.69–0.99) within PFP and healthy participants. More specifically, the MyJump2 App performed marginally better compared to Tracker across all variables (r=0.83–0.97 vs r=0.83–0.89 respectively). In our study, the MyJump2 App typically underestimated jump height, a similar outcome to that of Balsalobre-Fernández et al.^[9]^ However, in our study, the MyJump2 App marginally overestimated contact time and underestimated flight time, which was not the case with Balsalobre-Fernández et al.^[9]^, who found near-perfect agreements between the two apparatus. Tracker appeared to exhibit some selective bias at higher values across all parameters than the force plates. Such a finding may be attributed to the lower sampling frequency of the cell phone device (60Hz), which may introduce some artefact that affects precise parameter estimation within clinical settings. These results contrast with a study using the Digital Motion Analysis System, another computer-based video analysis system, where near-perfect agreement (r=0.988) was observed for jump height compared to the force plate measurement.^[18]^ The latter study, however, used substantially lower sampling frequencies for their force plate system (500Hz) and higher sampling frequencies for their video-based system (80Hz), which likely over-inflates their level of agreement and accounts for the observed discrepancies. Greater errors are possible because of the speed at which the video can be recorded on a cell phone, where there is a chance that the take-off and/or landing frame may not be recorded, resulting in less accurate contact- and flight times.^[9]^ More explicitly, an error of a single frame at take-off and landing, respectively (i.e. two total frames) can result in an error of ~6.0cm when recording at 60Hz.

The calculations based on the marker trajectories from the mocap system also provided higher jump height and flight time values than the force plates, but nearly identical values for contact time. The discrepancies associated with marker-based tracking (i.e., mocap and Tracker) may be due to the imprecision in isolating the exact take-off and landing time points due to differences in sampling frequencies.^[19]^ For example, an inaccuracy of just two frames for defining take-off and landing when recording at 60Hz can result in a measurement error in the jump height of ~0.06 m. It is for this reason that the general recommendation for the use of the MyJump2 App is a frame rate of 240Hz for optimal precision in the jump height estimate.^[20]^

Regarding jump height assessment, all alternative technologies compared favourably with force plates, typically yielding a mean error of 1–2cm, with the MyJump2 App exhibiting the narrowest margin of error. The ease of use and the narrow margin of error would position the MyJump2 App as the tool of choice for evaluating jump height in the sample evaluated. However, important differences in contact time were evident based on the wider margin of error, which would have implications for extracting key metrics such as the reactive strength index (RSI). This provides an avenue for future research. Although the MyJump2 App did have a small mean bias compared to force plates, the wider margin of error may have implications for evaluating those with PFP. Finally, mean bias and margin of error for flight time were comparable across devices, with Tracker showing the most favourable approximations compared to force plates. It is, however, important to mention that the substantially more time-consuming nature of data extraction and parameter calculations associated with Tracker compared to MyJump2 App (e.g. 5–10 orders of magnitude) likely limits the utility of the app, especially within clinical settings.

### Limitations

Although this study had several benefits, there were also a few limitations. Given that the present study sought to validate measurement tools, participants (especially those with PFP) could follow any rehabilitation program with any practitioner of their choice. Therefore, there was no control over the exercises prescribed. Future studies should, therefore, attempt to control the specific intervention programme to determine its effect on the measured parameters. Further, the ratio of males versus females differed between the control and PFP groups, although no other anthropometric differences were apparent. Lastly, the analysis of variables using the Tracker software was time-consuming, which may discourage practitioners from using the software despite the open-access nature of the tool.

## Conclusion

This study aimed to evaluate the validity of low-cost devices during unilateral jumping relative to ‘gold standard’ equipment and determine differences in kinematic variables between low-cost devices across the different groups. We found that kinematic parameters can be extracted from low-cost tools such as video and cell phone applications. The MyJump2 App was found to provide the greatest validity, especially for jump height and flight time metrics. This, coupled with its ease of use, makes this an attractive tool for evaluating both healthy and injured groups.

## References

[1] Dey P, Callaghan M, Cook N (2016). A questionnaire to identify patellofemoral pain in the community: an exploration of measurement properties. BMC Musculoskel Disord.

[2] Kunene SH, Ramklass S, Taukobong NP (2018). Anterior knee pain and its intrinsic risk factors among runners in under-resourced communities in Ekurhuleni, Gauteng. S Afr J Physiother.

[3] Reijnders L, Van de Groes SAW (2020). The quality of life of patients with patellofemoral pain - a systematic review. Acta Orthop Belg.

[4] Thomas MJ, Wood L, Selfe J, Peat G Anterior knee pain in younger adults as a precursor to subsequent patellofemoral osteoarthritis: a systematic review. BMC Musculoskelet Disord.

[5] Hart HF, Patterson BE, Crossley KM (2022). May the force be with you: understanding how patellofemoral joint reaction force compares across different activities and physical interventions - a systematic review and meta-analysis. J Sports Med.

[6] Collins NJ, Bierma-Zeinstra SMA, Crossley KM, Van Linschoten RL, Vicenzino B, Van Middelkoop M (2013). Prognostic factors for patellofemoral pain: a multicentre observational analysis. Br J Sports Med.

[7] Conceição F, Lewis M, Lopes H, Fonseca EM (2022). An evaluation of the accuracy and precision of jump height measurements using different technologies and analytical methods. Appl Sci.

[8] Ancillao A, Tedesco S, Barton J, O’Flynn B (2018). Indirect measurement of ground reaction forces and moments by means of wearable inertial sensors: a systematic review. Sensors.

[9] Balsalobre-Fernández C, Glaister M, Lockey RA (2015). The validity and reliability of an iPhone app for measuring vertical jump performance. J Sports Sci.

[10] Amoroso A, Rinaudo M (2018). Study of oscillatory motion using smartphones and tracker software. Institute of Physics Publishing Conference Series.

[11] Christensen LB, Johnson RB, Turner LA, Christensen LB, Johnson RB, Turner LA (2015). Chapter 8: Creating the Appropriate Research Design. Research methods, design and analysis.

[12] Faul F, Erdfelder E, Lang A, Buchner A (2007). G*Power 3: A flexible statistical power analysis program for the social, behavioral, and biomedical sciences. Behav Res Methods.

[13] Wang LI, Peng HT (2014). Biomechanical comparisons of single- and double-legged drop jumps with changes in drop height. Int J Sports Med.

[14] Schober P, Boer C, Schwarte LA (2018). Correlation coefficients: appropriate use and interpretation. Anesth Analg.

[15] Hair J, Ringle C, Sarstedt M (2011). PLS-SEM: Indeed a silver bullet. J Mark Theory Pract.

[16] Giavarina D (2015). Understanding bland altman analysis. Biochem Med (Zagreb).

[17] Hebbali A olsrr: Tools for Building OLS Regression Models. R package [software online, version 0.6.1].

[18] Dias JA, Pupo JD, Reis DC (2011). Validity of two methods for estimation of vertical jump height. J Strength Cond Res.

[19] Watkins C, Maunder E, Tillaar R (2020). Concurrent validity and reliability of three ultra-portable vertical jump assessment technologies. Sensors.

[20] Pueo B, Hopkins WG, Penichet-Tomas A, Jimenez-Olmedo J (2023). Accuracy of flight time and countermovement-jump height estimated from videos at different frame rates with MyJump. Biol Sport.

